# Plasma microbial cell-free DNA characterization in different populations based on the droplet digital PCR method: a multi-cohort study

**DOI:** 10.3389/fmicb.2025.1578820

**Published:** 2025-04-29

**Authors:** Juan Du, Dong Zhang, Fengliu Wang, Xuesong Shang, Jingjia Zhang, Guanhua Wang, Jingwen Ding, Aiwu Ren, Shuang Jing, Lu Bai, Ying Liu, Ye Zhao, Peng Li, Shuyi Yang, Jing Liu, Xuefang Xiang, Qiang Chen, Yingchun Xu, Jiang Xia, Qiwen Yang

**Affiliations:** ^1^Department of Clinical Laboratory, Peking Union Medical College Hospital, Peking Union Medical College and Chinese Academy of Medical Sciences, Beijing, China; ^2^Key Laboratory of Pathogen Infection Prevention and Control, Peking Union Medical College, Ministry of Education, Beijing, China; ^3^Pilot Gene Technologies Co., Ltd., Hangzhou, China

**Keywords:** plasma microbial cell-free DNA, bloodstream infection, sepsis, pathogen, droplet digital PCR

## Abstract

**Background:**

Plasma microbial cell-free DNA (mcfDNA) is a key biomarker for diagnosing bloodstream infections (BSIs), which contribute significantly to morbidity and mortality, particularly in patients with severe trauma, chronic illnesses, or immunosuppressive conditions. However, the baseline distribution of mcfDNA in different populations remains unclear. This study characterizes plasma mcfDNA profiles across various human populations.

**Methods:**

A total of 300 blood samples were collected from 10 groups: healthy individuals (Group A), patients with chronic diseases but no infections (Group B1–B7), patients with mild-to-moderate infections (Group C), and patients meeting sepsis criteria (Group D). Multiplex droplet digital PCR (ddPCR) was used to detect mcfDNA from 10 common sepsis-causing bacterial species, two fungal species, and three herpesviruses (HSV-1, Epstein–Barr virus [EBV], and Cytomegalovirus [CMV]).

**Results:**

Most pathogens in all groups showed low mcfDNA copy concentrations (~100 copies/mL), forming a baseline. Group A had no pathogens exceeding this level, while Group B showed elevated *E. coli* and *S. maltophilia* (10^2^–10^4^ copies/mL). In Group C, 53 pathogens were detected above baseline, with EBV, CMV, and HSV-1 as the most common (copy concentrations 10^2^–10^4^ copies/mL). In Group D, 57 pathogens exceeded baseline, primarily EBV, *K. pneumoniae*, *A. baumannii*, *E. faecium*, and CMV. Although statistical analysis showed no significant differences in pathogen distribution between Groups C and D, Gram-negative bacteria were more prevalent in Group D (70% vs. 53.3%, OR = 2.03), while viral pathogens were more frequently detected in Group C (93.3% vs. 76.7%, OR = 0.24). The microbial profiles and mcfDNA copy concentrations in Groups C and D were similar (10^2^–10^4^ copies/mL), distinguishing them from Groups A and B.

**Conclusion:**

This study provides a comprehensive characterization of mcfDNA across different health states, demonstrating the utility of ddPCR in detecting microbial infections. These findings contribute to refining infection diagnostics and improving early detection strategies for BSIs and sepsis.

## Introduction

Cell-free DNA (cfDNA) in plasma originates from senescent/apoptotic cells or invasive microorganisms that release nucleic acids into the blood during breakdown (Lee et al., 2020). Endogenous cfDNA has evolved into an indispensable biomarker in clinical practice for rapid and noninvasive diagnosis in liquid biopsy, noninvasive prenatal screening (NIPT), medication guidance, and tumor monitoring (Decker and Sholl, 2019; Sun et al., 2019; Liu et al., 2019; Wu et al., 2020; Liang et al., 2019). Exogenous cfDNA can be used to identify microbial pathogens invading the body under pathological conditions, such as bacteremia, viremia, and sepsis. The positive rate of microbial cell-free DNA (mcfDNA) detection is higher compared to that of traditional microbial detection. Currently, mcfDNA plays an important role in the diagnosis and treatment of clinical infections, such as sepsis, community-acquired pneumonia, tuberculosis, invasive mycosis, urinary tract infection, and infection after organ transplantation (Fernández-Carballo et al., 2019; Hong et al., 2018; Long et al., 2016; Weerakoon and McManus, 2016). In a high-throughput sequencing study of plasma mcfDNA, 348 patients with suspected sepsis were included in clinical practice, and their positive coincidence rate was 93.7% compared to the first positive blood culture. Among patients who developed antibiotic resistance before the onset of symptoms, the proportion of reliable or confirmed pathogens detected by next-generation sequencing technology (NGS) was 47.9%, and the sensitivity of blood culture was 19.6%, indicating the advantage of mcfDNA in detecting microbial infections (Blauwkamp et al., 2019). In the mcfDNA detection of pathogens in sepsis, Gram-negative bacteria accounted for the majority, followed by Gram-positive bacteria, fungi, and viruses. The most common Gram-negative bacteria isolates were *Klebsiella pneumoniae* (*K. pneumoniae*), *Escherichia coli* (*E. coli*), *Acinetobacter baumannii* (*A. baumannii*), and *Pseudomonas aeruginosa* (*P. aeruginosa*). The most frequent Gram-positive bacteria isolates were *Staphylococcus aureus* (*S. aureus*), Group B *Streptococcus* (GBS), *Coagulase-negative Staphylococci* (CoNS), and *Enterococcus* spp. (Kern and Rieg, 2020; Wu et al., 2022; Wang et al., 2021). The detection frequency of fungi is relatively lower than that of bacteria and viruses, with the main strains being *Candida tropicalis* (*C. tropicali*) and *Candida albicans* (*C. albicans*) (Wu et al., 2022; Wang et al., 2021). In terms of virus type, *herpes simplex virus–1* (HSV–1), *Cytomegalovirus* (CMV), and *Epstein–Barr virus* (EBV) accounted for the most cases (Wang et al., 2021; Cao et al., 2022).

The pathogens causing sepsis vary greatly depending on the infection source, patient characteristics, and disease course. For instance, from the results of three independent studies in China, the predominant pathogen for neonatal early-onset-sepsis (EOS) was *E. coli* (Mariani et al., 2022; Zou et al., 2021; Jiang et al., 2019), whereas in Italy, CoNS represented the predominant pathogen (Liu et al., 2021). A study of 9,381 bloodstream infection (BSI) episodes collected from hospitalized adult patients revealed that the composition ratios of CoNS, *E. coli*, and *K. pneumoniae* dynamically increased, whereas the proportion of *P. aeruginosa* decreased in China from 2010 to 2019 (Cui et al., 2022). In Australia, *Viridans streptococci* and *Enterococcus* species were the most predominant Gram-positive bacteria in neutropenic patients with hematological malignancies (Carvalho et al., 2020). In Chinese patients with solid tumors, the most common Gram-positive bacteria were *Staphylococcus aureus* and *Enterococcus* spp. (Xue et al., 2023).

Conventional blood culture (BC) is the gold standard for BSI diagnosis; however, it has several disadvantages, including long turnaround times, limited sensitivity, risk of contamination, and the inability to identify some fastidious organisms (Ehren et al., 2019; Kim et al., 2021; Sinha et al., 2018; Peker et al., 2018). Therefore, many culture-independent detection systems for BSI have rapidly been developed (Blauwkamp et al., 2019; Wu et al., 2022; Zboromyrska et al., 2019; Clancy et al., 2018; Nguyen et al., 2019; Yanagihara et al., 2010). Among these methods, the third-generation PCR after real-time quantitative PCR (qPCR), droplet digital PCR (ddPCR), has emerged as a promising and reliable tool for microorganism detection that takes advantage of high sensitivity, high precision, and absolute quantification (Merino et al., 2022; Luo et al., 2019; Li et al., 2019; Adjorlolo and Egbenya, 2020). The basic principle of ddPCR is to divide a PCR reaction system containing an enzyme and buffer, nucleic acid templates, primers, and probes into tens to hundreds of thousands of droplet units using microfluidic chips. A small percentage of the droplet unit contains one or more copies of nucleic acid molecules (DNA, RNA, or cDNA) and performs the PCR reactions independently. After routine PCR amplification, concentrations are determined based on the proportion of non-fluorescent partitions by Poisson distribution (Kojabad et al., 2021). ddPCR exhibits excellent performance in detecting bacteria, fungi, and viruses. In 2019, the sensitivity and specificity of the ddPCR method for tuberculosis (TB) detection were 95.7 and 88.9%, respectively (Luo et al., 2019). In the diagnosis of invasive fungal infection (IFI) in neonates, ddPCR had both high specificity (100%) and sensitivity (3.2 copies/μL) (Li et al., 2019). Compared to RT-qPCR, RT-ddPCR can detect SARS-CoV-2 at as low as 11.1 copies per test, which is 11 times lower than the number of 123.3 copies observed by RT-qPCR (Adjorlolo and Egbenya, 2020). Most reports on BSI pathogens have focused on a single group of people, such as neonates or the elderly, or people with specific clinical conditions, including cancer and trauma (Hu et al., 2021; Yan et al., 2021; Costescu Strachinaru et al., 2021; Lin et al., 2023). In this study, we characterized the mcfDNA profiles of different populations with healthy status, underlying chronic disease, local mild-to-moderate infections, and severe sepsis, with the aim to broaden the understanding of mcfDNA from different physical states and clarify the use of ddPCR in detecting infections.

## Materials and methods

### Study population and sample collection

This study was conducted during the period between August 13, 2021 and September 10, 2022, and was approved by Peking Union Medical College Hospital (PUMCH). Informed consent was obtained directly from the patients or their relatives for sample collection. The study enrolled 300 adult patients, including healthy people and patients with different underlying diseases or suspected BSI. The study population was classified into 10 Groups (Group A, B1–B7, C, D) and 30 candidates were recruited for each cohort. In total, 300 blood samples were collected and examined. Group A represented healthy people with normal physical examination results; Group B represented people who had underlying diseases (diabetes [B1], immunosuppression [B2], lung cancer [B3], breast cancer [B4], gastric cancer [B5], colorectal cancer [B6], and liver cancer [B7]) but were clinically free of infection; Group C represented patients with confirmed local infection but without BSI; and Group D represented patients who met the Sepsis 3.0 criteria and had a high probability of BSI. Patients with mental disorders and pregnant women were excluded.

An additional tube of 5 mL EDTA-anticoagulated peripheral blood was drawn simultaneously when blood cultures were performed on clinical indications (i.e., at the discretion of the attending physician) using the same venipuncture site for collecting a Cell-Free DNA BCT device (Streck, Nebraska US). The centrifugation procedure (1,600 g for 15 min at 4°C) was performed within 4 h of blood sample collection, and the plasma was separated for nucleic acid extraction and testing. If the sample could not be centrifuged in time, it was stored at 4°C for a maximum of 3 days before further processing. Samples were anonymized for patient characteristics and the corresponding culture results using coded identifiers.

### Nucleic acid extraction

The plasma was separated for nucleic acid extraction. Peripheral blood specimens were centrifuged at 1600 g for 15 min at 4°C. Subsequently, 1 mL plasma and 10 μL internal control were mixed evenly and transferred to an Auto-Pure 10B nucleic acid purification system (Hangzhou Allsheng Instruments Co., Ltd., Hangzhou, China) for cfDNA isolation using a Magnetic Serum/Plasma DNA Kit (Tiangen Biotech, Beijing, China) according to the manufacturer’s instructions. The cfDNA was eluted into 60 μL of 10 mM Tris-EDTA buffer and stored at −80°C until final analysis.

### Droplet digital PCR

The multiplex ddPCR assay enables the simultaneous detection of common bacterial pathogens, fungal pathogens, viruses (detailed information in the below table) directly from blood samples. ddPCR analysis was performed using a 5-fluorescent-channel droplet digital PCR system (Pilot Gene Technology Co., Ltd., Hangzhou, China). Briefly, 5 μL cfDNA template was added to 10 μL ddPCR premix, including the detection primers, probes, and necessary components for PCR amplification. The synthesized DNA fragments served as positive controls, and DNase-free water served as negative controls to eliminate external or reagent microbial contamination. The reaction mixture was gently mixed and added to a ready-to-use disposable plastic chip. Approximately 20,000 water-in-oil emulsion droplets were generated inside the chip using a droplet generator (DG32, Pilot Gene Tech.). The chips were then amplified in a thermal cycler (TC1, Pilot Gene Tech.) using the following cycling parameters: 95°C for 5 min, followed by 40 cycles of 95°C for 15 s and 60°C for 60 s. Finally, the chips were loaded into a chip scanner (iScanner 5, Pilot Gene Tech.) for fluorescence signal reading and further data analysis. The data were analyzed using the GeneDPT software (Pilot Gene Tech.). According to the manufacturer’s instructions for the assay panels, the threshold for target detection was 0.5 copies/μL. ddPCR was defined as positive when the concentration exceeded the threshold.

**Table:** 

Panel	FAM	VIC	ROX	CY5
1	*P. aeruginosa*	*E. coli*	*K. pneumoniae*	*A. baumannii*
2	*S. aureus*	*E. faecium*	*E. faecalis*	*S. pneumoniae*
3	*S. maltophilia*	*E. cloacae*	*S. marcescens*	*P. mirabilis*
4	*S. haemolyticus*	*S. hominis*	*S. capitis*	*S. epidermidis*
5	*C. albican*	*C. glabrata*	*C. parapsilosis*	*C. tropicalis*
6	*HSV-1*	*VZV*	*EBV*	*CMV*

### Blood culture (BC)

Blood culture at PUMCH was processed using the BC instrument BACTEC FX TOP (BD, USA). Positive blood culture samples were subcultured on blood agar plates, and microorganism colonies were identified using Vitek MS (bioMérieux, France) through matrix-assisted laser desorption/ionization time-of-flight mass spectrometry (MALDI-TOF).

### Ethics approval and participation

The human research ethics committee of the Institutional Review Board of Peking Union Medical College Hospital approved this study. This project did not affect the normal diagnosis and treatment of patients. Formal ethics approval was obtained and waived, and written patient consent was not required after consultation with the Institutional Review Board (ethics approval number HS-2952).

### Statistical analysis

Continuous variables are expressed as the median and interquartile range (IQR). Figures were created using Prism 8.4.0 software (GraphPad Software, San Diego, CA, USA).

## Results

### Characteristics of the populations

Positive cases detected by digital PCR were predominantly enrolled from intensive care units (ICUs), including the stroke intensive care unit (SICU), emergency intensive care unit (EICU), medical intensive care unit (MICU), respiratory intensive care unit (RICU), and emergency internal medicine. The clinical characteristics of the recruited patients are detailed in Table 1. The target microbial pathogens included Gram-negative bacteria (*P. aeruginosa, E. coli, K. pneumoniae, A. baumannii, P. mirabilis, S. aureus, E. faecium*), Gram-positive bacteria (*S. maltophilia, E. cloacae, S. epidermidis*), fungi (*C. albicans, C. parapsilosis*), and herpesviruses (HSV-1, EBV, CMV).

**Table 1:** Clinical characteristics of the recruited patients.

Clinical characteristics		Group A	Group B	Group C	Group D
Demographic characteristics	Age, years	37.4 ± 11.6	56.3 ± 12.5	56.9 ± 20.1	61.5 ± 16.41
	Male, *n* (%)	18 (60)	106 (51.2)	22 (73.3)	21 (71)
Department or type of disease		Medical examination center: 30	Medical oncology: 70	ICU: 7	ICU: 2
			Hematology and oncology: 30	EICU: 2	ED: 8
			Department of rheumatology and immunology: 24	SICU: 10	MICU: 2
			Department of endocrinology: 18	MICU: 7	Emergency internal medicine: 7
			Other: 64	RICU: 4	Other: 11
Laboratory examination	WBC count, median (IQR) × 10^3^/μL	5.49 (4.68–6.06)	5.48 (4.38–7.27)	13.07 (5.83–17.90)	10.01 (6.69–13.19)
	CRP (mg/L), median (IQR)	/	1.86 (0.6–5.49)	135.85 (69.33–169.05)	131.35 (52.7–191.6)
	PCT (μg/L), median (IQR)	/	/	2.5 (1.36–6.2)	2.4 (0.36–20)

ICU, intensive care unit; EICU, emergency intensive care unit; SICU, stroke intensive care unit; MICU, medical intensive care unit; RICU, respiratory intensive care unit; ED, emergency department; WBC, white blood cell; IQR, interquartile range; CRP, C-reactive protein; PCT, procalcitonin.

### Copy concentrations of bacterial pathogens in different populations

**Figure 1:**
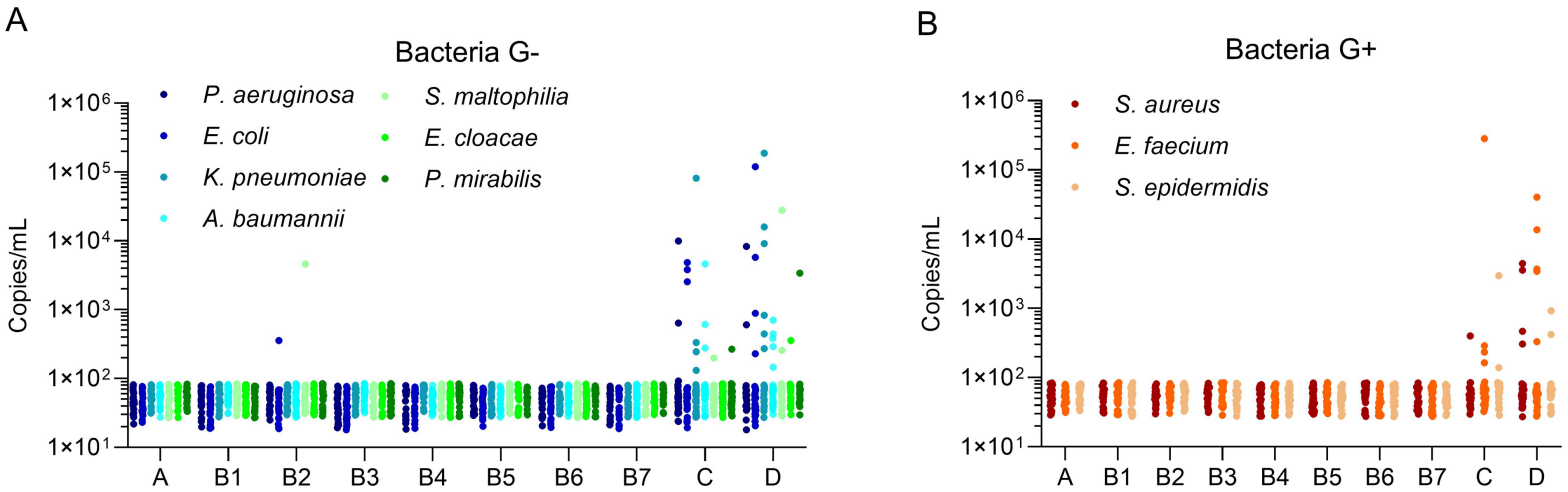
Microbial cell-free DNA levels of bacterial pathogens in different populations. **(A)** Microbial cell-free DNA levels of Gram negative bacteria. **(B)** Microbial cell-free DNA levels of Gram positive bacteria. The Y-axis indicated the copy concentration (copies/mL). The X-axis indicated patient groups. Bacteria G+, Gram positive bacteria, Bacteria G–, Gram negative bacteria.

For Gram-negative bacteria (Figure 1A), the mcfDNA copy concentration of most pathogens in the 10 groups was relatively low, forming a baseline (~100 copies/mL). There were very few pathogens exceeding the baseline level in Groups A and B, whereas there were more pathogens in Groups C and D. The mcfDNA copy concentrations (above the baseline) in Groups C and D were commonly distributed between 10^2^ and 10^4^ copies/mL. There was only one target (*K. pneumoniae*) with a copy concentration of 10^4^–10^5^ copies/mL in Group C, whereas in Group D, there were two targets (*K. pneumoniae*, *S. maltophilia*) with concentrations of 10^4^–10^5^ copies/mL and two targets (*K. pneumoniae*, *E. coli*) with concentrations of 10^5^–10^6^ copies/mL. For Gram-positive bacteria (Figure 1B), the trend of copy concentration was similar to that in Figure 1A. The mcfDNA copy concentrations of bacterial pathogens in Groups A and B was ≤ 100 copies/mL, whereas the copy concentrations in Groups C and D were commonly distributed between 10^2^ and 10^4^ copies/mL. One target (*S. epidermidis*) in Group C had a copy concentration > 10^5^ copies/mL, whereas another target (*E. faecium*) had copy concentrations of 10^4^–10^5^ copies/mL in Group D. Infections with *E. coli* and *S. maltophilia* (10^2^–10^4^copies/mL) occurred in Group B2, which was expected given that the candidates in Group B2 represented immunocompromised patients who were easily infected during treatment. The total number of bacterial infections was higher in Group D than in Group C, demonstrating their classification consistency; that is, the severity of sepsis was higher in Group D than in Group C. Notably, there was no significant difference in the distribution of copy concentration between the two groups. In some severe cases, the copy concentration even reached 10^5^ copies/mL. Group C included patients with confirmed infection symptoms but without sepsis (verified by blood culture), but multiple pathogens were still detected in the blood, indicating that patients in Group C may be in the early stage of sepsis and require timely treatment.

### Copy concentrations of fungi and viruses in different populations

**Figure 2:**
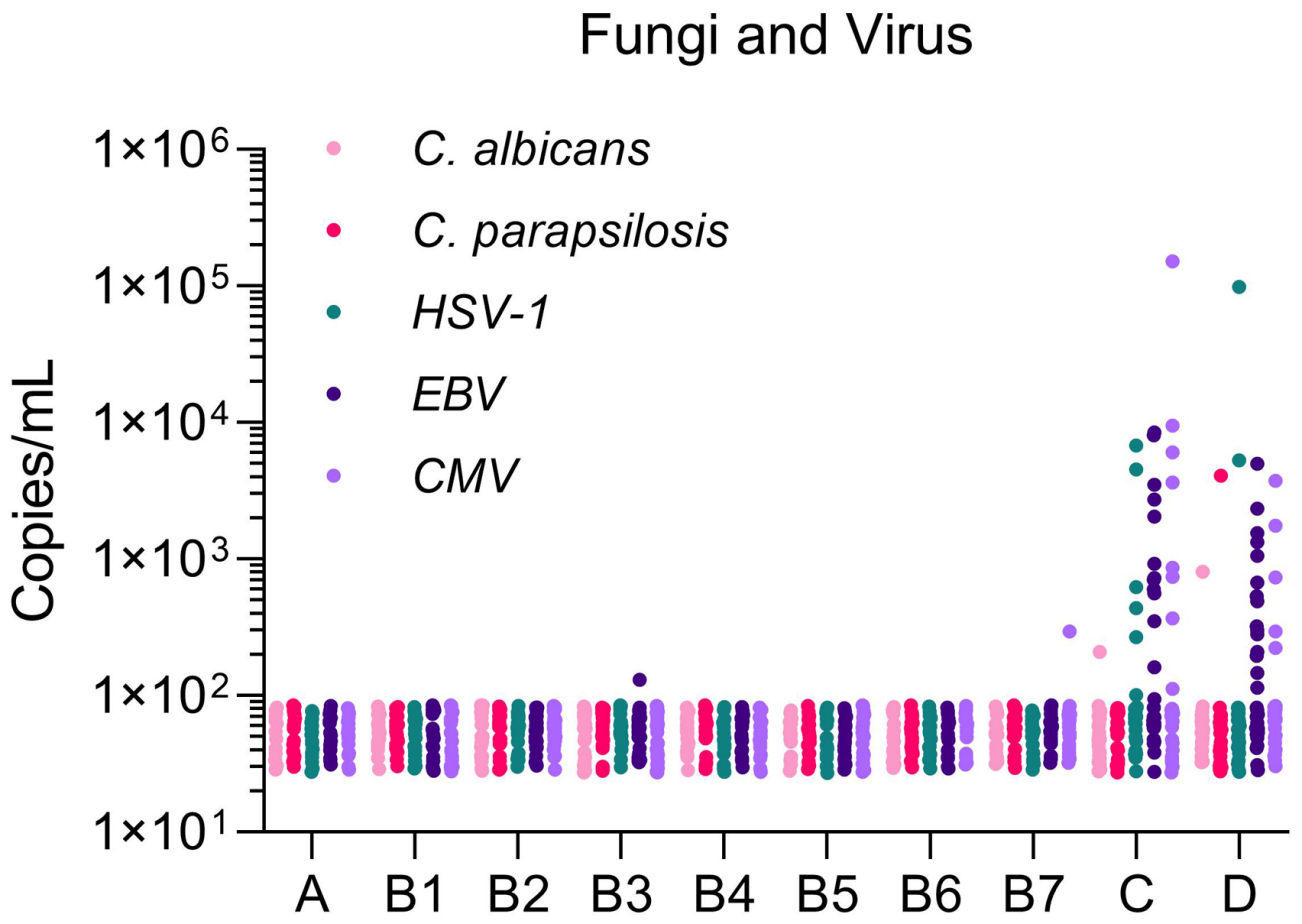
Microbial cell-free DNA levels of fungi and virus in different populations. The Y-axis indicated the copy concentration (copies/mL). The X-axis indicated patient groups.

In this study, two fungi (*C. albicans*, *C. parapsilosis*) and three herpesviruses (HSV-1, EBV, CMV) were detected. Most mcfDNA copy concentrations of fungi/herpesviruses were ≤ 100 copies/mL in Groups A and B. Some infections (EBV in Group B3 and CMV in Group B7) still existed in Group B, but the copy concentration was lower than 300 copies/mL. The copy concentrations of the fungi were commonly distributed between 100 and 1,000 copies/mL in Groups C and D. For herpesviruses in Groups C/D, the range of copy concentrations was 10^2^–10^4^ copies/mL. One pathogen copy concentration exceeded 10^4^ copies/mL in Groups C and D, respectively (Figure 2). In general, the baseline mcfDNA copy concentration is similar to that of bacteria. The number of viral infections was significantly higher than that of fungal infections.

**Figure 3:**
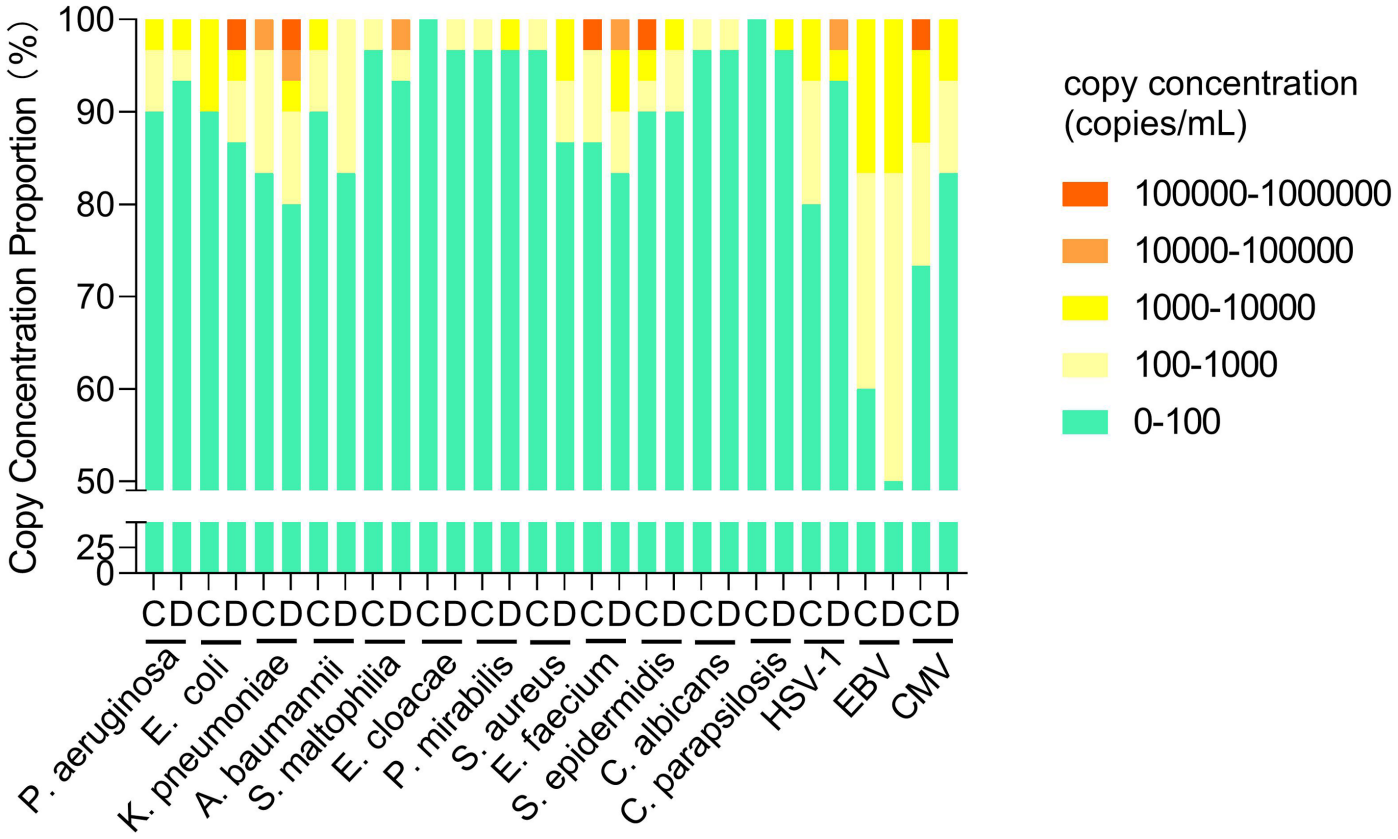
Comparison of the mcfDNA copy concentration of target pathogens in groups C and D. mcfDNA, microbial cell-free DNA.

We next analyzed the mcfDNA copy concentration of target pathogens in five gradients for Groups C and D. The proportions in Groups C and D were consistent for most pathogens at 10^2^–10^4^ copies/mL, except for *A. baumannii, S. aureus, HSV-1, EBV,* and *CMV*, whose proportions differed in Groups C and D (10% vs. 16.67, 3.33% vs. 13.34, 20% vs. 3.33, 40% vs. 50, 23.33% vs. 16.67%, respectively). Above 10^4^ copies/mL, no significant difference was observed in the proportion between Groups C and D, but the copy concentration varied. For *E. coli, K. pneumoniae, S. maltophilia*, and *HSV–1*, the copy concentration in Group D was higher, whereas for *E. faecium, S. epidermidis,* and *CMV*, Group C had a higher concentration (Figure 3).

### mcfDNA profiles in Groups C and D

Because > 97% of positive episodes appeared in Groups C and D, we focused on the analysis of these two groups. No significant differences were observed between the two groups regarding gender (χ^2^ = 0.093, *p* = 0.761) or age (*U* = 406.5, *p* = 0.798), demonstrating comparable baseline demographics (Table 2). A total of 53 and 57 pathogens were detected in Groups C and D, respectively. The statistical analysis of positive case counts for target pathogens in both groups is presented in Table 3. Both the Fisher–Freeman–Halton test (*p* = 0.892) and the chi-square test (*p* = 0.775) demonstrated no significant differences in pathogen distribution between the two groups. Although *S. aureus* (OR = 4.46) and *A. baumannii* (OR = 1.67) showed higher detection rates in Group D, these differences did not reach statistical significance (all *p* > 0.05). Viral pathogens (EBV/CMV/HSV) exhibited similar detection rates between two groups, suggesting potential latent infections. The most frequently detected Gram-negative bacteria in both groups were *K. pneumoniae, A. baumannii, E. coli, P. aeruginosa* while the predominant Gram-positive bacteria were *S. aureus* and *E. faecium*, which is consistent with the CHINET surveillance data. Although statistical analysis showed no significant differences in pathogen distribution between Groups C and D, Gram-negative bacteria were more prevalent in Group D (70% vs. 53.3%, OR = 2.03), while viral pathogens were more frequently detected in Group C (93.3% vs. 76.7%, OR = 0.24) (Table 4). More than 35% of infections were caused by a single pathogen (36.7% for Group C and 50% for Group D). In addition to polymicrobial infections, two or three types of pathogens account for the majority (Table 5).

**Table 2:** Clinical characteristics by cohort.

Variable	Group C (*n* = 30)	Group D (*n* = 30)	Statistic	*p* value
Male sex (*n*, %)	22 (73.3%)	21 (70%)	χ^2^ = 0.093	0.761
Age (years)	66 (34–71)	63 (55–68)	U = 406.5	0.798

Data are median (IQR).

**Table 3:** Comparison of pathogen species between Group C and D.

Pathogen type	Group C (*n* = 30)	Group D (*n* = 30)	*p* value	OR (95%CI)
*P. aeruginosa*	3	2	1.000	0.64 (0.10–4.06)
*E. coli*	3	4	1.000	1.33 (0.27–6.56)
*K. pneumoniae*	5	6	1.000	1.20 (0.34–4.27)
*A. baumannii*	3	5	0.706	1.67 (0.37–7.55)
*S. maltophilia*	1	2	1.000	2.00 (0.17–23.1)
*E. cloacae*	0	1	1.000	/
*P. mirabilis*	1	1	1.000	1.00 (0.06–16.3)
*S. aureus*	1	4	0.358	4.46 (0.47–42.3)
*E. faecium*	5	5	1.000	1.00 (0.26–3.80)
*S. epidermidis*	2	2	1.000	1.00 (0.13–7.54)
*C. albicans*	1	1	1.000	1.00 (0.06–16.3)
*C. parapsilosis*	0	1	1.000	/
*HSV-1*	6	3	0.476	0.47 (0.11–2.02)
*EBV*	14	15	1.000	1.14 (0.42–3.12)
*CMV*	8	5	0.542	0.58 (0.17–2.00)

**Table 4:** Comparison of pathogen types between Group C and D.

Pathogen type	Group C (*n* = 30)	Group D (*n* = 30)	*p* value	OR (95%CI)
G−	16 (53.3%)	21 (70%)	0.34	2.03 (0.76–5.45)
G+	8 (26.7%)	11 (36.7%)	0.59	1.61 (0.55–4.70)
Fungi	1 (3.3%)	2 (6.7%)	1.00	2.06 (0.18–24.1)
Virus	28 (93.3%)	23 (76.7%)	0.11	0.24 (0.05–1.22)

G−, Gram-negative bacteria; G+, Gram-positive bacteria; OR, odds ratio.

**Table 5:** Counts of polymicrobial infections between Group C and D.

Pathogen type	Group C (*n* = 30)	Group D (*n* = 30)	*p* value	OR (95%CI)
0	6 (20%)	2 (6.7%)	0.25	0.29 (0.05–1.59)
1	11 (36.7%)	15 (50%)	0.43	1.73 (0.62–4.84)
2	7 (23.3%)	5 (16.7%)	0.75	0.66 (0.18–2.41)
3	3 (10%)	7 (23.3%)	0.30	2.80 (0.64–12.3)
≥4	3 (10%)	1 (3.3%)	0.61	0.31 (0.03–3.20)

OR, odds ratio.

### Agreement analysis between ddPCR findings and clinical diagnostic outcomes

A comparison between ddPCR and blood culture results in Groups C and D demonstrated high sensitivity (>93%) for ddPCR. In Group D, ddPCR failed to detect two blood culture-positive targets: one case of possible blood culture contamination (*S. hominis*), and one confirmed missed detection of *S. aureus*, suggesting room for further optimization of the ddPCR assay. For Group C, ddPCR detected 16 additional positive cases compared to blood culture, highlighting its superior sensitivity (Table 6). However, the lack of validation by a third diagnostic method represents a limitation of this study.

**Table 6:** Positive and negative agreement of ddPCR versus BC, microbiological testing and clinical diagnosis.

Sample (*n* = 60)		Diagnosis+	Diagnosis-	Sensitivity (%)	Specificity (%)	PPV (%)	NPV (%)
Total	ddPCR+	36	16	94.74 (82.25–99.36)	27.27 (10.73–50.22)	69.23 (54.90–81.28)	75 (34.91–96.81)
	ddPCR-	2	6				
Group C	ddPCR+	8	16	100 (63.06–100)	27.27 (10.73–50.22)	33.33 (15.63–55.32)	100 (54.07–100)
	ddPCR-	0	6				
Group D	ddPCR+	28	0	93.33	/	100	/
	ddPCR-	2	0				

PPV, positive predictive value; NPV, negative predictive value.

## Discussion

In this study, we identified 10 bacterial species involved in the most common “ESKAPE” pathogens (*E. coli, S. aureus, K. pneumoniae, A. baumannii, P. aeruginosa, E. faecium*), as well as two fungi species (*C. albicans, C. parapsilosis*) and three herpesviruses (HSV-1, EBV, and CMV) in four groups. None of the target pathogens were found in healthy subjects (group A). The incidence of BSI in patients with cancer or immunosuppression (group B) was low (1.4%), representing 3 of 210 episodes. Group C consisted of patients with confirmed infection symptoms but without BSI; Group D consisted of patients with a SOFA (Sequential Organ Failure Assessment) score increment ≥ 2 or qSOFA ≥ 2 (prophase) and a high probability of BSI. The distribution and mcfDNA copy concentrations of microbial pathogens in Groups C and D were similar. The high detection of mcfDNA in the blood stream of patients with symptoms of infection but without BSI and those with a high likelihood of BSI suggested that mcfDNA in plasma originated from invasive microorganisms, which release nucleic acids into the blood as they breakdown. The microbial pathogen profiles of BSI in different populations provide a reliable basis for clinical treatment.

ddPCR, as an emerging detection tool, has many advantages, such as high sensitivity and accurate quantification of pathogen nucleic acids, and can be used for a variety of purposes. ddPCR has the advantage of absolute quantification in the field of dynamic monitoring, which NGS cannot achieve. mcfDNA can be continuously detected in patients with bloodstream infection or sepsis and evaluated through dynamic monitoring (Kustanovich et al., 2019). A prospective pilot study showed that mcfDNA-seq has the potential to predict most episodes of BSI before onset in high-risk pediatric cancer patients. Moreover, mcfDNA-seq can predict infections in approximately 75% of relapsed pediatric cancer patients with impending BSI, with a specificity of > 80%. In a 2019 study, the sensitivity and specificity of the ddPCR method for TB detection were 95.7 and 88.9%, respectively (Goggin et al., 2020). From this perspective, ddPCR with high sensitivity and specificity can also be used as a dynamic monitoring test for mcfDNA in patients with BSI.

However, ddPCR still has some limitations. Studies have shown that the plasma cfDNA concentration of healthy people fluctuates between 0.1 and 1.5 ng/μL, and human DNA accounts for the majority (> 90% or even > 99%), whereas microbial DNA accounts for only a small proportion (Blauwkamp et al., 2019). cfDNA mainly arises from the degradation of genomic DNA of senescent and apoptotic cells under normal physiological conditions, whereas under pathological conditions, such as malignant tumors, trauma, organ transplant rejection, autoimmune diseases, and sepsis, a large amount of cfDNA can be released into the bloodstream by abnormal necrotic cells. Thus, the origin of cfDNA in plasma is complex, and while ddPCR can detect mcfDNA in plasma, it is unable to distinguish mcfDNA between living and apoptotic microorganisms. However, this is not an important constraint for the application of ddPCR, as other molecular diagnostic methods, such as qPCR and metagenomics-based assays, are also unable to distinguish this situation.

Definition of the constant value/cut-off value of the target pathogenetic organisms detected by molecular diagnostic methods to provide a basis for clinical diagnosis still requires a combination of the clinical background of patients, imaging data, and other laboratory examinations. Although ddPCR can quantify the copy concentration of plasma cfDNA, there is still no uniform standard for the specific positive values of different diseases and target pathogens. Similar to mNGS, there is currently no accepted threshold, and the methods/standards for dividing the thresholds vary widely between studies. For patients with suspected BSI, the levels of procalcitonin (PCT) and CPR (C-reactive protein) can be used to evaluate the degree of bacterial infection and prognosis. In fact, in addition to laboratory tests, the copy concentration level can reflect the severity of infection and provide medication guidance. For example, Hirsch et al. (2005) reported that a plasma BK virus (BKV) DNA level > 4 log10 copies/mL was recommended for a presumed diagnosis of BKVAN (BKV-associated nephropathy), and the urinary viral load in affected patients exceeded 7 log10 copies/mL. The Kidney Disease: Improving Global Outcomes (KDIGO) clinical practice guidelines also suggest a reduction in immunosuppression when the BKV load in plasma is persistently > 4 log10 copies/mL to prevent disease progression to an irreversible phase (Eckardt and Kasiske, 2009). In addition, according to the European Conference on Infections in Leukemia guidelines, one of the “diagnostic triad” for BKV-related hemorrhagic cystitis is BKV viruria ≥ 7 log10 copies/mL (Cesaro et al., 2017). However, among the numerous studies on the pathogens causing BSI, most focused on the types of pathogens and did not analyze the correlation between quantitative ddPCR results and severity stratification of pathogen load. The copy concentrations of the microbial pathogens in our tests were generally distributed between 10^2^ and 10^4^ copies/mL. However, due to the lack of clinical information and the lack of comparison of blood culture results, it is not yet possible to establish an accurate relationship between copy concentration and disease severity.

Reactivated herpesviruses in septic patients during ICU stay mainly involve HSV-1, EBV, CMV, and HHV-6 (Human herpesvirus 6). Among these herpesviruses, EBV has the highest incidence. EBV reactivation in sepsis has been shown to be associated with an increased risk of mortality (Mallet et al., 2019; Ong et al., 2017) and was also observed in a diverse Group of ICU patients (Libert et al., 2015). Similarly, the incidence rate of EBV was close to 25%, which was higher than that of other bacterial or fungal pathogens in our research. Viral infection is gradually becoming an important factor in BSI and requires additional attention.

## Data Availability

The raw data supporting the conclusions of this article will be made available by the authors, without undue reservation.

## Glossary

*A. baumannii*: *Acinetobacter baumannii*
BC: Blood culture
BKV: BK virus
BKVAN: BKV-associated nephropathy
BSI: Bloodstream infection
*C. albicans*: *Candida albicans*
*C. tropicali*: *Candida tropicalis*
cfDNA: Cell-free DNA
CMV: Cytomegalovirus
CoNS: *Coagulase-negative Staphylococci*
CPR: C-reactive protein
ddPCR: Droplet digital PCR
*E. coli*: *Escherichia coli*
EBV: Epstein–Barr virus
EICU: Emergency intensive care unit
EOS: Early-onset-sepsis
GBS: Group B Streptococcus
HSV–1: Herpes simplex virus–1
ICU: Intensive care unit
IFI: Invasive fungal infection
IQR: Interquartile range
*K. pneumoniae*: *Klebsiella pneumoniae*
KDIGO: Kidney disease improving global outcomes
mcfDNA: Microbial cell-free DNA
MICU: Medical intensive care unit
NGS: Next-generation sequencing technology
NIPT: Noninvasive prenatal screening
*P. aeruginosa*: *Pseudomonas aeruginosa*
PCT: Procalcitonin
PUMCH: Peking Union Medical College Hospital
qPCR: Quantitative PCR
RICU: Respiratory intensive care unit
*S. aureus*: *Staphylococcus aureus*
SICU: Stroke intensive care unit
TB: Tuberculosis
HHV-6: Human herpesvirus 6
SOFA: Sequential organ failure assessment

## References

[ref1] Adjorlolo S. Egbenya D. L. (2020). A twin disaster: addressing the COVID-19 pandemic and a cerebrospinal meningitis outbreak simultaneously in a low-resource country. Glob. Health Action 13:1795963. doi: 10.1080/16549716.2020.1795963, PMID: 32762300 PMC7480482

[ref2] Blauwkamp T. A. Thair S. Rosen M. J. Blair L. Lindner M. S. Vilfan I. D.. (2019). Analytical and clinical validation of a microbial cell-free DNA sequencing test for infectious disease. Nat. Microbiol. 4, 663–674. doi: 10.1038/s41564-018-0349-6, PMID: 30742071

[ref3] Cao X.-G. Zhou S.-S. Wang C.-Y. Jin K. Meng H.-D. (2022). The diagnostic value of next-generation sequencing technology in sepsis. Front. Cell. Infect. Microbiol. 12:899508. doi: 10.3389/fcimb.2022.899508, PMID: 36189371 PMC9518011

[ref4] Carvalho A. S. Lagana D. Catford J. Shaw D. Bak N. (2020). Bloodstream infections in neutropenic patients with haematological malignancies. Infect Dis Health 25, 22–29. doi: 10.1016/j.idh.2019.08.006, PMID: 31586572

[ref5] Cesaro S. Dalianis T. Hanssen Rinaldo C. Koskenvuo M. Pegoraro A. Einsele H.. (2017). ECIL guidelines for the prevention, diagnosis and treatment of BK polyomavirus-associated haemorrhagic cystitis in haematopoietic stem cell transplant recipients. J. Antimicrob. Chemother. doi: 10.1093/jac/dkx324, PMID: 29190347

[ref6] Clancy C. J. Pappas P. G. Vazquez J. Judson M. A. Kontoyiannis D. P. Thompson G. R.. (2018). Detecting infections rapidly and easily for candidemia trial, part 2 (DIRECT2): a prospective, multicenter study of the T2Candida panel. Clin. Infect. Dis. 66, 1678–1686. doi: 10.1093/cid/cix1095, PMID: 29438475

[ref7] Costescu Strachinaru D. I. Gallez J.-L. Daras S. Paridaens M.-S. Engel H. François P.-M.. (2021). A case of *Flavonifractor plautii* blood stream infection in a severe burn patient and a review of the literature. Acta Clin. Belg. 77, 693–697. doi: 10.1080/17843286.2021.1944584, PMID: 34151750

[ref8] Cui J. Li M. Cui J. Wang J. Qiang X. Liang Z. (2022). The proportion, species distribution and dynamic trends of bloodstream infection cases in a tertiary hospital in China, 2010-2019. Infection 50, 121–130. doi: 10.1007/s15010-021-01649-y, PMID: 34184182 PMC8803777

[ref9] Decker B. Sholl L. M. (2019). Cell-free DNA testing. Genomic medicine. Cham: Springer, 41–54.

[ref10] Eckardt K.-U. Kasiske B. L. (2009). Kidney disease: improving global outcomes. Nat. Rev. Nephrol. 5, 650–657. doi: 10.1038/nrneph.2009.153, PMID: 19786993

[ref11] Ehren K. Meißner A. Jazmati N. Wille J. Jung N. Vehreschild J. J.. (2019). Clinical impact of rapid species identification from positive blood cultures with same-day phenotypic antimicrobial susceptibility testing on the management and outcome of bloodstream infections. Clin. Infect. Dis. 70, 1285–1293. doi: 10.1093/cid/ciz406, PMID: 31094414

[ref12] Fernández-Carballo B. L. Broger T. Wyss R. Banaei N. Denkinger C. M. (2019). Toward the development of a circulating free DNA-based *in vitro* diagnostic test for infectious diseases: a review of evidence for tuberculosis. J. Clin. Microbiol. 57, e01234–e01218. doi: 10.1128/JCM.01234-18, PMID: 30404942 PMC6440766

[ref13] Goggin K. P. Gonzalez-Pena V. Inaba Y. Allison K. J. Hong D. K. Ahmed A. A.. (2020). Evaluation of plasma microbial cell-free DNA sequencing to predict bloodstream infection in pediatric patients with relapsed or refractory cancer. JAMA Oncol. 6, 552–556. doi: 10.1001/jamaoncol.2019.4120, PMID: 31855231 PMC6990667

[ref14] Hirsch H. H. Brennan D. C. Drachenberg C. B. Ginevri F. Gordon J. Limaye A. P.. (2005). Polyomavirus-associated nephropathy in renal transplantation: interdisciplinary analyses and recommendations. Transplantation 79, 1277–1286. doi: 10.1097/01.TP.0000156165.83160.09, PMID: 15912088

[ref15] Hong D. K. Blauwkamp T. A. Kertesz M. Bercovici S. Truong C. Banaei N. (2018). Liquid biopsy for infectious diseases: sequencing of cell-free plasma to detect pathogen DNA in patients with invasive fungal disease. Diagn. Microbiol. Infect. Dis. 92, 210–213. doi: 10.1016/j.diagmicrobio.2018.06.009, PMID: 30017314

[ref16] Hu B. Tao Y. Shao Z. Zheng Y. Zhang R. Yang X.. (2021). A comparison of blood pathogen detection among droplet digital PCR, metagenomic next-generation sequencing, and blood culture in critically ill patients with suspected bloodstream infections. Front. Microbiol. 12:641202. doi: 10.3389/fmicb.2021.641202, PMID: 34079528 PMC8165239

[ref17] Jiang S. Hong L. Gai J. Shi J. Yang Y. Lee S. K.. (2019). Early-onset sepsis among preterm neonates in China, 2015 to 2018. Pediatr. Infect. Dis. J. 38, 1236–1241. doi: 10.1097/INF.0000000000002492, PMID: 31738341

[ref18] Kern W. V. Rieg S. (2020). Burden of bacterial bloodstream infection—a brief update on epidemiology and significance of multidrug-resistant pathogens. Clin. Microbiol. Infect. 26, 151–157. doi: 10.1016/j.cmi.2019.10.031, PMID: 31712069

[ref19] Kim J. H. Kim I. Kang C. K. Jun K. I. Yoo S. H. Chun J. Y.. (2021). Enhanced antimicrobial stewardship based on rapid phenotypic antimicrobial susceptibility testing for bacteraemia in patients with haematological malignancies: a randomized controlled trial. Clin. Microbiol. Infect. 27, 69–75. doi: 10.1016/j.cmi.2020.03.038, PMID: 32272171

[ref20] Kojabad A. A. Farzanehpour M. Galeh H. E. G. Dorostkar R. Jafarpour A. Bolandian M.. (2021). Droplet digital PCR of viral DNA/RNA, current progress, challenges, and future perspectives. J. Med. Virol. 93, 4182–4197. doi: 10.1002/jmv.26846, PMID: 33538349 PMC8013307

[ref21] Kustanovich A. Schwartz R. Peretz T. Grinshpun A. (2019). Life and death of circulating cell-free DNA. Cancer Biol. Ther. 20, 1057–1067. doi: 10.1080/15384047.2019.1598759, PMID: 30990132 PMC6606043

[ref22] Lee R. A. Al Dhaheri F. Pollock N. R. Sharma T. S. (2020). Assessment of the clinical utility of plasma metagenomic next-generation sequencing in a pediatric hospital population. J. Clin. Microbiol. 58, e00419–e00420. doi: 10.1128/JCM.00419-20, PMID: 32376666 PMC7315020

[ref23] Li H. T. Lin B. C. Huang Z. F. Yang C. Z. Huang W. M. (2019). Clinical value of droplet digital PCR in rapid diagnosis of invasive fungal infection in neonates. Chin J Contemp Pediatr 21, 45–51. doi: 10.7499/j.issn.1008-8830.2019.01.009 PMC7390180 30675863

[ref24] Liang W. Zhao Y. Huang W. Gao Y. Xu W. Tao J.. (2019). Non-invasive diagnosis of early-stage lung cancer using high-throughput targeted DNA methylation sequencing of circulating tumor DNA (ctDNA). Theranostics 9, 2056–2070. doi: 10.7150/thno.28119, PMID: 31037156 PMC6485294

[ref25] Libert N. Bigaillon C. Chargari C. Bensalah M. de Rudnicki S. Muller V.. (2015). Epstein-Barr virus reactivation in critically ill immunocompetent patients. Biom. J. 38, 70–76. doi: 10.4103/2319-4170.132905, PMID: 25179711

[ref26] Lin K. Zhao Y. Xu B. Yu S. Fu Z. Zhang Y.. (2023). Clinical diagnostic performance of droplet digital PCR for suspected bloodstream infections. Microbiol Spectr. 11:e0137822. doi: 10.1128/spectrum.01378-22, PMID: 36602351 PMC9927361

[ref27] Liu Y. Fan Z. Zhou Y. Lin J. Yang Y. Yan L.. (2019). Self-circulating electrochemiluminescence chip for sensitive detection of circulating tumour nucleic acids in blood. Sensors Actuators B Chem. 301:127088. doi: 10.1016/j.snb.2019.127088

[ref28] Liu J. Fang Z. Yu Y. Ding Y. Liu Z. Zhang C.. (2021). Pathogens distribution and antimicrobial resistance in bloodstream infections in twenty-five neonatal intensive care units in China, 2017–2019. Antimicrob. Resist. Infect. Control 10:121. doi: 10.1186/s13756-021-00989-6, PMID: 34399840 PMC8365905

[ref29] Long Y. Zhang Y. Gong Y. Sun R. Su L. Lin X.. (2016). Diagnosis of sepsis with cell-free DNA by next-generation sequencing technology in ICU patients. Arch. Med. Res. 47, 365–371. doi: 10.1016/j.arcmed.2016.08.004, PMID: 27751370

[ref30] Luo J. Luo M. Li J. Yu J. Yang H. Yi X.. (2019). Rapid direct drug susceptibility testing of *Mycobacterium tuberculosis* based on culture droplet digital polymerase chain reaction. Int. J. Tuberc. Lung Dis. 23, 219–225. doi: 10.5588/ijtld.18.0182, PMID: 30808455

[ref31] Mallet F. Perret M. Tran T. Meunier B. Guichard A. Tabone O.. (2019). Early herpes and TTV DNAemia in septic shock patients: a pilot study. Intensive Care Med. Exp. 7:28. doi: 10.1186/s40635-019-0256-z, PMID: 31104220 PMC6525672

[ref32] Mariani M. Parodi A. Minghetti D. Ramenghi L. A. Palmero C. Ugolotti E.. (2022). Early and late onset neonatal sepsis: epidemiology and effectiveness of empirical antibacterial therapy in a III level neonatal intensive care unit. Antibiotics 11:284. doi: 10.3390/antibiotics11020284, PMID: 35203886 PMC8868064

[ref33] Merino I. de la Fuente A. Domínguez-Gil M. Eiros J. M. Tedim A. P. Bermejo-Martín J. F. (2022). Digital PCR applications for the diagnosis and management of infection in critical care medicine. Crit. Care 26:63. doi: 10.1186/s13054-022-03948-8, PMID: 35313934 PMC8935253

[ref34] Nguyen M. H. Clancy C. J. Pasculle A. W. Pappas P. G. Alangaden G. Pankey G. A.. (2019). Performance of the T2Bacteria panel for diagnosing bloodstream infections. Ann. Intern. Med. 170, 845–852. doi: 10.7326/M18-2772, PMID: 31083728

[ref35] Ong D. S. Y. Bonten M. J. M. Spitoni C. Verduyn Lunel F. M. Frencken J. F. Horn J.. (2017). Epidemiology of multiple herpes viremia in previously immunocompetent patients with septic shock. Clin. Infect. Dis. 64, 1204–1210. doi: 10.1093/cid/cix120, PMID: 28158551

[ref36] Peker N. Couto N. Sinha B. Rossen J. W. (2018). Diagnosis of bloodstream infections from positive blood cultures and directly from blood samples: recent developments in molecular approaches. Clin. Microbiol. Infect. 24, 944–955. doi: 10.1016/j.cmi.2018.05.007, PMID: 29787889

[ref37] Sinha M. Jupe J. Mack H. Coleman T. P. Lawrence S. M. Fraley S. I. (2018). Emerging technologies for molecular diagnosis of sepsis. Clin. Microbiol. Rev. 31, e00089–e00017. doi: 10.1128/CMR.00089-17, PMID: 29490932 PMC5967692

[ref38] Sun Y. An K. Yang C. (2019). “Circulating cell-free DNA” in Liquid Biopsy. eds. Strumfa I. Gardovskis J. (London: Intech Open).

[ref39] Wang L. Guo W. Shen H. Guo J. Wen D. Yu Y.. (2021). Plasma microbial cell-free DNA sequencing technology for the diagnosis of sepsis in the ICU. Front. Mol. Biosci. 8:659390. doi: 10.3389/fmolb.2021.659390, PMID: 34124149 PMC8194294

[ref40] Weerakoon K. G. McManus D. P. (2016). Cell-free DNA as a diagnostic tool for human parasitic infections. Trends Parasitol. 32, 378–391. doi: 10.1016/j.pt.2016.01.006, PMID: 26847654

[ref41] Wu J. Hu S. Zhang L. Xin J. Sun C. Wang L.. (2020). Tumor circulome in the liquid biopsies for cancer diagnosis and prognosis. Theranostics 10, 4544–4556. doi: 10.7150/thno.40532, PMID: 32292514 PMC7150480

[ref42] Wu J. Tang B. Qiu Y. Tan R. Liu J. Xia J.. (2022). Clinical validation of a multiplex droplet digital PCR for diagnosing suspected bloodstream infections in ICU practice: a promising diagnostic tool. Crit. Care 26:243. doi: 10.1186/s13054-022-04116-8, PMID: 35941654 PMC9358819

[ref43] Xue L. Zhu Y. Zong M. Jiao P. Fu J. Liang X.-M.. (2023). Clinical characteristics of bloodstream infections in adult patients with solid tumours and a nomogram for mortality prediction: a 5-year case-controlled retrospective study in a tertiary-level hospital. Front. Cell. Infect. Microbiol. 13:13. doi: 10.3389/fcimb.2023.1228401, PMID: 37614558 PMC10442815

[ref44] Yan G. Liu J. Chen W. Chen Y. Cheng Y. Tao J.. (2021). Metagenomic next-generation sequencing of bloodstream microbial cell-free nucleic acid in children with suspected sepsis in pediatric intensive care unit. Front. Cell. Infect. Microbiol. 11:665226. doi: 10.3389/fcimb.2021.665226, PMID: 34504805 PMC8421769

[ref45] Yanagihara K. Kitagawa Y. Tomonaga M. Tsukasaki K. Kohno S. Seki M.. (2010). Evaluation of pathogen detection from clinical samples by real-time polymerase chain reaction using a sepsis pathogen DNA detection kit. Crit. Care 14:R159. doi: 10.1186/cc9234, PMID: 20731880 PMC2945143

[ref46] Zboromyrska Y. Cillóniz C. Cobos-Trigueros N. Almela M. Hurtado J. C. Vergara A.. (2019). Evaluation of the Magicplex™ sepsis real-time test for the rapid diagnosis of bloodstream infections in adults. Front. Cell. Infect. Microbiol. 9:56. doi: 10.3389/fcimb.2019.00056, PMID: 30931259 PMC6423426

[ref47] Zou H. Jia X. He X. Su Y. Zhou L. Shen Y.. (2021). Emerging threat of multidrug resistant pathogens from neonatal sepsis. Front. Cell. Infect. Microbiol. 11:11. doi: 10.3389/fcimb.2021.694093, PMID: 34322398 PMC8312093

